# Ethnicity, consanguinity, and genetic architecture of hypertrophic cardiomyopathy

**DOI:** 10.1093/eurheartj/ehad372

**Published:** 2023-07-11

**Authors:** Mona Allouba, Roddy Walsh, Alaa Afify, Mohammed Hosny, Sarah Halawa, Aya Galal, Mariam Fathy, Pantazis I Theotokis, Ahmed Boraey, Amany Ellithy, Rachel Buchan, Risha Govind, Nicola Whiffin, Shehab Anwer, Ahmed ElGuindy, James S Ware, Paul J R Barton, Magdi Yacoub, Yasmine Aguib

**Affiliations:** Aswan Heart Centre, Magdi Yacoub Heart Foundation, Kasr El Haggar Street, Aswan 81512, Egypt; National Heart and Lung Institute, Imperial College London, London, Guy Scadding Building, Dovehouse St, London SW3 6LY, UK; National Heart and Lung Institute, Imperial College London, London, Guy Scadding Building, Dovehouse St, London SW3 6LY, UK; Department of Experimental Cardiology, Amsterdam UMC, University of Amsterdam, Meibergdreef 9, Amsterdam 1105 AZ, The Netherlands; Aswan Heart Centre, Magdi Yacoub Heart Foundation, Kasr El Haggar Street, Aswan 81512, Egypt; Aswan Heart Centre, Magdi Yacoub Heart Foundation, Kasr El Haggar Street, Aswan 81512, Egypt; Cardiology Department, Kasr Al Aini Medical School, Cairo University, Kasr Al Aini Street, Cairo 11562, Egypt; Aswan Heart Centre, Magdi Yacoub Heart Foundation, Kasr El Haggar Street, Aswan 81512, Egypt; Aswan Heart Centre, Magdi Yacoub Heart Foundation, Kasr El Haggar Street, Aswan 81512, Egypt; Aswan Heart Centre, Magdi Yacoub Heart Foundation, Kasr El Haggar Street, Aswan 81512, Egypt; National Heart and Lung Institute, Imperial College London, London, Guy Scadding Building, Dovehouse St, London SW3 6LY, UK; Aswan Heart Centre, Magdi Yacoub Heart Foundation, Kasr El Haggar Street, Aswan 81512, Egypt; Cardiology Department, Kasr Al Aini Medical School, Cairo University, Kasr Al Aini Street, Cairo 11562, Egypt; Aswan Heart Centre, Magdi Yacoub Heart Foundation, Kasr El Haggar Street, Aswan 81512, Egypt; National Heart and Lung Institute, Imperial College London, London, Guy Scadding Building, Dovehouse St, London SW3 6LY, UK; Royal Brompton & Harefield Hospitals, Guy’s and St. Thomas’ NHS Foundation Trust, London, Sydney St, London SW3 6NP, UK; National Heart and Lung Institute, Imperial College London, London, Guy Scadding Building, Dovehouse St, London SW3 6LY, UK; Royal Brompton & Harefield Hospitals, Guy’s and St. Thomas’ NHS Foundation Trust, London, Sydney St, London SW3 6NP, UK; Present affiliation: Institute of Psychiatry, Psychology and Neuroscience, King's College London, 16 De Crespigny Park, London SE5 8AF, UK; Present affiliation: National Institute for Health Research (NIHR) Biomedical Research Centre, South London and Maudsley NHS Foundation Trust and King's College London, 16 De Crespigny Park, London SE5 8AF, UK; National Heart and Lung Institute, Imperial College London, London, Guy Scadding Building, Dovehouse St, London SW3 6LY, UK; Royal Brompton & Harefield Hospitals, Guy’s and St. Thomas’ NHS Foundation Trust, London, Sydney St, London SW3 6NP, UK; Present affiliation: Wellcome Centre for Human Genetics, University of Oxford, Roosevelt Dr, Headington, Oxford OX3 7BN, UK; Aswan Heart Centre, Magdi Yacoub Heart Foundation, Kasr El Haggar Street, Aswan 81512, Egypt; Aswan Heart Centre, Magdi Yacoub Heart Foundation, Kasr El Haggar Street, Aswan 81512, Egypt; National Heart and Lung Institute, Imperial College London, London, Guy Scadding Building, Dovehouse St, London SW3 6LY, UK; Royal Brompton & Harefield Hospitals, Guy’s and St. Thomas’ NHS Foundation Trust, London, Sydney St, London SW3 6NP, UK; MRC London Institute of Medical Sciences, Imperial College London, Du Cane Rd, London W12 0NN, UK; National Heart and Lung Institute, Imperial College London, London, Guy Scadding Building, Dovehouse St, London SW3 6LY, UK; Royal Brompton & Harefield Hospitals, Guy’s and St. Thomas’ NHS Foundation Trust, London, Sydney St, London SW3 6NP, UK; MRC London Institute of Medical Sciences, Imperial College London, Du Cane Rd, London W12 0NN, UK; Aswan Heart Centre, Magdi Yacoub Heart Foundation, Kasr El Haggar Street, Aswan 81512, Egypt; National Heart and Lung Institute, Imperial College London, London, Guy Scadding Building, Dovehouse St, London SW3 6LY, UK; Harefield Heart Science Centre, Hill End Rd, Harefield, Uxbridge UB9 6JH, UK; Aswan Heart Centre, Magdi Yacoub Heart Foundation, Kasr El Haggar Street, Aswan 81512, Egypt; National Heart and Lung Institute, Imperial College London, London, Guy Scadding Building, Dovehouse St, London SW3 6LY, UK

**Keywords:** Egyptian collaborative cardiac genomics project, Homozygosity, NGS

## Abstract

**Aims:**

Hypertrophic cardiomyopathy (HCM) is characterized by phenotypic heterogeneity that is partly explained by the diversity of genetic variants contributing to disease. Accurate interpretation of these variants constitutes a major challenge for diagnosis and implementing precision medicine, especially in understudied populations. The aim is to define the genetic architecture of HCM in North African cohorts with high consanguinity using ancestry-matched cases and controls.

**Methods and results:**

Prospective Egyptian patients (*n* = 514) and controls (*n* = 400) underwent clinical phenotyping and genetic testing. Rare variants in 13 validated HCM genes were classified according to standard clinical guidelines and compared with a prospective HCM cohort of majority European ancestry (*n* = 684). A higher prevalence of homozygous variants was observed in Egyptian patients (4.1% vs. 0.1%, *P* = 2 × 10^−7^), with variants in the minor HCM genes *MYL2*, *MYL3*, and *CSRP3* more likely to present in homozygosity than the major genes, suggesting these variants are less penetrant in heterozygosity. Biallelic variants in the recessive HCM gene *TRIM63* were detected in 2.1% of patients (five-fold greater than European patients), highlighting the importance of recessive inheritance in consanguineous populations. Finally, rare variants in Egyptian HCM patients were less likely to be classified as (likely) pathogenic compared with Europeans (40.8% vs. 61.6%, *P* = 1.6 × 10^−5^) due to the underrepresentation of Middle Eastern populations in current reference resources. This proportion increased to 53.3% after incorporating methods that leverage new ancestry-matched controls presented here.

**Conclusion:**

Studying consanguineous populations reveals novel insights with relevance to genetic testing and our understanding of the genetic architecture of HCM.

## Structured Graphical Abstract

**Structured Graphical Abstract:**
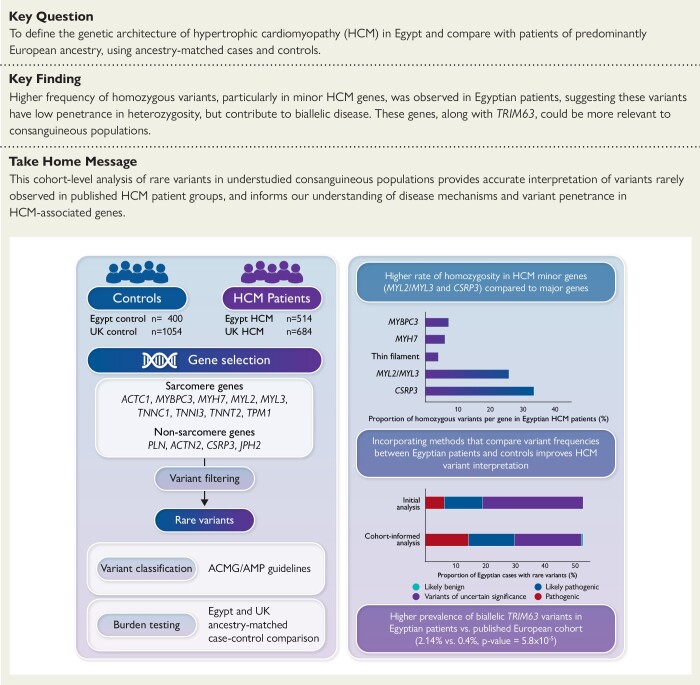
The genetic architecture of hypertrophic cardiomyopathy (HCM) in the Egyptian population was assessed by comparing the frequency of rare variation in HCM genes with Egyptian controls as well as with a UK HCM cohort (left). HCM minor genes MYL2, MYL3 and CSRP3 were more likely to present in homozygosity than the major genes, suggesting these variants are less penetrant in heterozygosity (top right). Integrating methods that leverage Egyptian controls increased the yield of clinically actionable variants (pathogenic, likely pathogenic) from 19% (initial analysis) to 29.6% (cohort-informed analysis) (bottom right). HCM: Hypertrophic Cardiomyopathy; ACMG/AMP: American College of Medical Genetics and Genomics/Association of Molecular Pathology. Created with BioRender.com.

**See the editorial comment for this article ‘The moral and practical urgency of increasing diversity in genomics’, by J. Ingles and D.G. MacArthur, https://doi.org/10.1093/eurheartj/ehad365.**

**Translational perspective**

This study comprehensively analyses the genetic architecture of hypertrophic cardiomyopathy (HCM) in underrepresented North African cohorts and identifies important contributors of disease unique to consanguineous populations. The analysis enables more accurate interpretation of variants rarely observed in published European-centric HCM patient groups. We also describe the characteristics of homozygous variants in dominant HCM genes, rarely observed in outbred populations. These findings will enhance the utility of clinical genetic testing for understudied populations and our understanding of the genetic aetiology of HCM. Analysis of such highly consanguineous cohorts opens new research avenues for exploring the genetic architecture of cardiac diseases like HCM.

## Introduction

Hypertrophic cardiomyopathy (HCM) is a common inherited cardiac condition that is estimated to affect 1:500 according to published studies.^1,2^ It is defined by left ventricular hypertrophy (LVH) in the absence of other identifiable cardiac or systemic causes. Hypertrophic cardiomyopathy has been considered as a highly heterogeneous disease with regard to mode of inheritance, phenotype, and genotype.^3–5^ The genetic aetiology of HCM is largely attributed to variation in genes that encode components of the contractile apparatus (sarcomere) in cardiac muscle cells.

Large HCM data sets from the Sarcomeric Human Cardiomyopathy Registry (SHaRe) and other diverse ancestral HCM populations have provided strong evidence supporting disease-associated, long-term adverse clinical outcomes, including mortality.^6–11^ With respect to genotype, SHaRe demonstrated that patients with pathogenic sarcomere variants had an earlier onset of disease and were at a higher risk of developing adverse clinical outcomes.^10^ These patients may benefit from effective treatments that delay or prevent HCM-related complications provided these pathogenic variants are well characterized.^10,12^ Moreover, the rapid integration of genetic testing into everyday clinical practice has resulted in the identification of a large number of variants of uncertain significance, which cannot be acted upon clinically.^10,13,14^ This issue is more evident in underrepresented populations, who are more likely to receive inconclusive genetic testing results, compared with well-studied European-ancestry HCM patients.^15^ Therefore, extending genomic research to diverse ancestral populations will not only reduce health disparities as we move towards precision-based HCM patient management but may also enhance variant interpretation for diagnosis and provide novel genetic and mechanistic insights into the disease. For example, we have recently reported the identification of a new class of HCM pathogenic variants in *MYH7*, which was enriched in Egyptian patients over ancestry-matched controls and absent from large-scale HCM patients of majority European ancestry.^16–18^

The aim of this study is to define the genetic architecture of HCM in the previously understudied Egyptian population that is known to have a high rate of consanguinity.^19,20^ This study provides, to our knowledge, the largest and most comprehensive analysis of HCM in the Middle East and North Africa (MENA) region to date. We leverage a prospective Egyptian HCM cohort and an ancestry-matched, Egyptian healthy volunteer control cohort with comprehensive cardiovascular phenotyping, to define the genetic architecture of HCM in Egypt. In addition, we compare genetic data between Egyptian and well-characterized patients of majority European ancestry to identify genetic features unique to consanguineous populations of MENA ancestry which are likely to be important contributors of HCM.

## Methods

### Study population

#### Egypt hypertrophic cardiomyopathy cohort

A prospective cohort of unrelated HCM index patients (*n* = 514), denoted hereafter as Egypt HCM, was recruited to the Aswan Heart Centre (AHC), Egypt, as part of the National HCM Registry and the Egyptian Collaborative Cardiac Genomics (ECCO-GEN) Project.^17^ Clinical diagnosis of HCM was confirmed by echocardiography and/or cardiac magnetic resonance (CMR) imaging. Consanguinity (self-reported) was defined as first-cousin marriage.

#### UK hypertrophic cardiomyopathy cohort

A prospective cohort of HCM index patients (*n* = 684), denoted hereafter as UK HCM, was recruited by the NIHR Cardiovascular Biomedical Research Unit at the Royal Brompton Hospital (RBH), London.^21^ Hypertrophic cardiomyopathy diagnosis was confirmed with CMR. The ethnicities of these patients were self-reported and they were of majority European ancestry (73.1% self-reported as European).

#### Egypt control cohort

Egyptian healthy volunteers (*n* = 400), denoted hereafter as Egypt controls, were recruited from across the country as part of the ECCO-GEN. All volunteers underwent detailed phenotyping, including clinical examination, 12-lead electrocardiogram, and CMR to confirm absence of cardiovascular disease. Principal component analysis (PCA) showed that both Egypt HCM patients and controls overlapped into one distinct cluster confirming that they are ancestry-matched (see Supplementary material online, *Figure S1*).

#### UK control cohort

UK healthy volunteers (*n* = 1054) of majority European ancestry, denoted hereafter as UK controls, were recruited prospectively via advertisement for the UK Digital Heart Project at Imperial College London.^22^ All controls underwent CMR to confirm the absence of cardiac disease.

Participants from all four cohorts provided informed consent and were approved by their local research ethics committees.

### Gene selection

DNA samples from cases and controls were sequenced on Illumina platforms and a subset of these on Life Technologies SOLiD 5500xl using cardiac gene panels including the TruSight Cardio Sequencing kit^23^ (see Supplementary material online, *Table S1*). Thirteen validated HCM genes that are usually associated with autosomal dominant inheritance, including sarcomere-encoding (*ACTC1*, *MYBPC3*, *MYH7*, *MYL2*, *MYL3*, *TNNC1*, *TNNI3*, *TNNT2*, and *TPM1*) and non–sarcomere-encoding genes (*PLN*, *ACTN2*, *CSRP3*, and *JPH2*), were analysed.^21,24,25^ Genes which have been previously shown to account for <5% of HCM cases were defined here as ‘minor’ genes.^18^  *MYH7* and *MYBPC3* were defined here as ‘major’ genes as they account for over 70% of identified pathogenic variants in HCM.^26^ Separately, biallelic variants in the recently implicated recessive HCM gene *TRIM63* were evaluated.

### Rare variant filtering and classification

Rare variants in HCM genes were defined as having a popmax filtering allele frequency (FAF) of ng × 10^−5^ in gnomAD (v2.1.1).^27^ ‘Popmax’ represents the highest FAF across the five main gnomAD subpopulations. Variants were annotated by CardioClassifier based on rules defined by the American College of Medical Genetics and Genomics/Association for Molecular Pathology (ACMG/AMP) as pathogenic (P), likely pathogenic (LP), and of uncertain significance (VUS) (see Supplementary material online, *Methods*).^28,29^ Detailed lists of all rare variants observed in the analysed cohorts are provided in Supplementary material online, *Tables S2–S5*.

### Comparison of the genetic landscape of hypertrophic cardiomyopathy between Egypt and UK hypertrophic cardiomyopathy cohorts

The genetic architecture of HCM in the Egyptian population was assessed by comparing the frequency of rare variation in HCM genes between ancestry-matched patients and controls for both the Egyptian and UK cohorts using Fisher’s exact tests (see Supplementary material online, *Tables S6* and *S7*). The genes and variant classes significantly enriched in cases were then compared between cohorts. To account for the overall differences in genetic yield between the two cohorts, the proportion of genotype-positive patients (i.e. those carrying rare variants) attributable to each gene was also compared between both cohorts.

### Comparison of the proportion of clinically actionable variants between the Egypt and UK hypertrophic cardiomyopathy cohorts

The proportion of clinically actionable variants (P/LP) was evaluated in the Egypt HCM cohort by dividing the number of patients with P/LP variants by the total number of patients with rare variants in HCM genes. For patients with multiple hits (see Supplementary material online, *Tables S8* and *S9*), the variant with the highest pathogenicity was prioritized (i.e. P then LP then VUS). The proportion of clinically actionable variants was then compared between the Egypt and UK HCM cohorts.

### Application of the new etiological fraction–based American College of Medical Genetics and Genomics/Association for Molecular Pathology rule on rare MYH7 missense variants

The effect of the recently proposed etiological fraction (EF)-based ACMG/AMP rules on the yield of actionable *MYH7* missense variants identified in the Egypt HCM cohort was assessed.^13^

First, we evaluated whether the regional distributions of pathogenic and benign variation in *MYH7*, previously observed in individuals of majority European ancestry, are also observed in Egyptians. The frequency of rare missense *MYH7* variants in the predefined patient-enriched HCM cluster (residues: 167–931) was calculated.^13^ The distribution of these variants in the Egypt HCM and control cohorts was assessed and compared with the previously published cohort of >6000 HCM patients from Partners Laboratory of Molecular Medicine and Oxford Medical Genetics Laboratory (denoted here as LMM/OMGL).^18^ Second, the odds ratio (OR) and EF (EF = (OR − 1)/OR) for this cluster were calculated using the Egypt control cohort as the reference population (see Supplementary material online, *Methods* for full details). *MYH7* missense variants were classified as per the adopted new EF-based PM1_strong rule (nontruncating variant in gene/protein region with EF ≥ 0.95), which is activated if these variants are located in a protein region with EF ≥ 0.95.^13^

## Results

### Cohort characteristics

The Egypt HCM cohort comprised 514 patients (67% male and 34% female; details in Supplementary material online, *Table S10*). Consanguinity was reported in 28.4% of patients with available family data. Notably, UK patients (*n* = 684) were ∼20 years older, presented with less severe hypertrophy, and showed a lower incidence of left ventricular outflow tract obstruction (LVOTO) compared with Egyptian patients (*Table 1*).

**Table 1:** Demographic and cardiac characteristics of Egypt and UK HCM cohorts

General characteristics	Egypt HCM	UK HCM	*P*-value
Demographics			
Age (years) [mean (SD)]	34.7 ± 17.4	55.5 ± 15.5	<2.2 × 10^−16^
Male sex	345 (67.3%)	473 (73.4%)	0.02
Offspring of consanguineous marriage^a^	99 (28.4%)	N/A	N/A
FH of HCM	111 (25.1%)	127 (24.5%)	0.88
FH of SCD	78 (17.6%)	116 (25.2%)	0.01
Cardiac characteristics			
LVMWT (mm)^b^ [mean (SD)]	23.6 ± 7	18.9 ± 4.4	4.9 × 10^−15^
Extreme LVMWT >30 mm	54 (17.5%)	8 (1.3%)	<2.2 × 10^−16^
LVOTO ≥30 mmHg	220 (72.8%)	223 (36.1%)	<2.2 × 10^−16^
LVEF (%)^b^ [mean (SD)]	71.5 ± 13.3	74.3 ± 8.5	0.004
Myectomy	168 (51.5%)	N/A	N/A
ICD implantation	7 (2.7%)	N/A	N/A

HCM, hypertrophic cardiomyopathy; FH, family history; SCD, sudden cardiac death; LVMWT, left ventricular maximal wall thickness; LVOTO, left ventricular outflow tract obstruction; LVEF, left ventricular ejection fraction; ICD, implantable cardioverter-defibrillator; N/A, not available; SD, standard deviation.

^a^Consanguinity (self-reported) was defined as first-cousin marriage.

^b^LVMWT and LVEF were measured in the Egypt HCM and UK HCM cohorts by echocardiography and CMR, respectively.

### Differences in the genetic architecture of hypertrophic cardiomyopathy between Egyptian and UK patients

**Figure 1:**
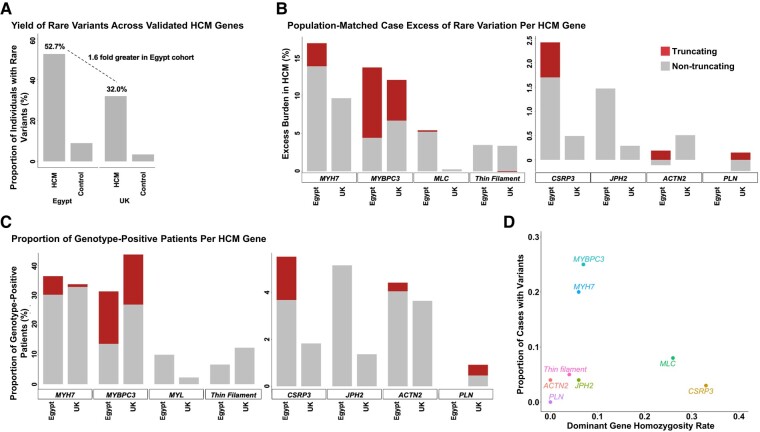
Enrichment of rare variation and homozygosity in hypertrophic cardiomyopathy minor genes (MLC and CSRP3) in the Egypt hypertrophic cardiomyopathy cohort vs. UK hypertrophic cardiomyopathy. (*A*) The overall frequency of rare variants (gnomAD_popmax_ FAF ≤4 × 10^−5^) in 13 validated hypertrophic cardiomyopathy genes was compared between Egypt and UK hypertrophic cardiomyopathy patients and controls. A 1.6-fold greater excess burden (i.e. total case frequency − total control frequency) was observed in the Egypt hypertrophic cardiomyopathy cohort, which should be accounted for with the direct gene comparisons (*B*). (*B*) Population-matched patients vs. control comparison (Egypt HCM_Control_ excess and UK HCM_Control_ excess) of genetic variation between Egypt and UK cohorts for each hypertrophic cardiomyopathy gene/gene class. (*C*) Comparison of the proportion of genotype-positive patients (i.e. those carrying rare variants) per hypertrophic cardiomyopathy gene confirms the enrichment of rare variants in the hypertrophic cardiomyopathy minor genes MLC (MYL2 and MYL3) and CSRP3 in Egyptians (as observed in *B*). Truncating and nontruncating variants (*A*, *B*) are shown in red and grey, respectively. Thin filament: ACTC1, TNNC1, TNNI3, TNNT2, and TPM1. (*D*) MLC (MYL2 and MYL3) and CSRP3 genes showed a markedly higher rate of homozygosity compared with major HCM genes (MYBPC3 and MYH7) and thin filament genes (ACTC1, TNNC1, TNNI3, TNNT2, and TPM1). HCM, hypertrophic cardiomyopathy.

We compared the burden of rare variation (regardless of zygosity) in HCM genes between Egyptian patients and controls (*Figure 1A*). The inclusion of ancestry-matched controls into this analysis enabled us to address the potential bias introduced by defining rare variants using gnomAD, which has minimal representation from the MENA region (see Supplementary material online, *Figure S2*).

The variant classes that are significantly enriched in cases for each cohort are shown in *Table 2* (full details in Supplementary material online, *Tables S6* and *S7*). We then compared the excess burden of rare variants in HCM genes in each cohort (labelled as Egypt HCM_Control_ excess and UK HCM_Control_ excess) (*Table 2*). A higher proportion of rare variation across all analysed HCM genes was observed in the Egypt HCM cohort compared with UK (52.7% vs. 32.0%) (*Figure 1A*), with 1.6-fold more Egyptian patients carrying rare variants compared with UK (*Figure 1A* and *B* and *Table 2*). Finally, to account for this overall difference in genetic yield, we also performed a secondary analysis comparing the proportion of genotype-positive patients (i.e. those carrying rare variants) attributable to each HCM gene between both ancestral cohorts (*Figure 1C*).

**Table 2:** Comparison of population-matched patient vs. control excess frequencies (left) and the proportion of genotype-positive patients between Egypt HCM and UK HCM cohorts (right) for sarcomeric and other validated HCM genes

Gene/gene groups	Variant type	Excess of RV in patients vs. controls		Proportion of all G+	
		Egypt HCM_Control_ excess	UK HCM_Control_ excess	Egypt HCM G+ prop	UK HCM G+ prop
*MYH7*	Nontruncating	13.95%**	9.68%**	30.26%	32.88%
	Truncating	3.06%**	0.01%	6.27%	0.91%
*MYBPC3*	Nontruncating	4.45%**	6.73%**	13.65%	26.94%
	Truncating	9.34%**	5.41%**	17.71%	16.89%
*Myosin light chain* (*MLC*)	Nontruncating	5.25%**	0.26%	9.96%	2.28%
	Truncating	0.19%	0.00%	0.37%	0%
*Thin Filament*	Nontruncating	3.50%**	3.38%**	6.64%	12.33%
	Truncating	0.00%	−0.09%	0%	0%
*CSRP3*	Nontruncating	1.70%*	0.49%	3.69%	1.83%
	Truncating	0.72%	0.00%	1.85%	0%
*JPH2*	Nontruncating	1.47%	0.29%	5.17%	1.37%
	Truncating	0%	0%	0%	0%
*ACTN2*	Nontruncating	−0.11%	0.51%	4.06%	3.65%
	Truncating	0.19%	0%	0.37%	0%
*PLN*	Nontruncating	0%	−0.23%	0%	0.46%
	Truncating	0%	0.15%	0%	0.46%

Comparison of the excess of rare (FAF ≤ 4 × 10^−5^) variation in HCM patients over controls for Egypt and UK cohorts, grouped by gene/gene class and variant class (left). Details of case–control comparisons are shown in Supplementary material online, *Table S6* (Egypt cohorts) and S7 (UK cohorts), summarized here as: enrichment in cases (Fisher’s exact one-sided test) with Bonferroni significance (**) or nominal significance (*). Note—the overall case excess across the validated HCM genes/gene classes is 1.6 times greater in the Egypt cohort (*Figure 1A*); therefore, any comparisons for specific genes or gene classes between Egypt and UK cohorts should take this into account. The proportion of genotype-positive (i.e. those with rare variants) Egypt HCM and UK HCM patients per HCM gene is shown (left).

RV, rare variants; G+, genotype-positive; prop, proportion.

As expected, rare variants in *MYH7* and *MYBPC3* accounted for the majority of rare variation in both cohorts (*Table 2*). *MYH7* truncating variants accounted for a higher proportion of genotype-positive patients in the Egypt HCM cohort, which was attributed to a single frameshift variant c.5769delG (p.Ser1924AlafsTer9) affecting 3.3% of patients.^16^ Several recurrent truncating variants in *MYBPC3* were also detected, with two of these c.1516delG (p.Asp506ThrfsTer7) (0.97%, *n* = 5) and c.534_541delGGCCGGCG (p.Ala179GlnfsTer59) (1.36%, *n* = 7) likely to be Egyptian-/North African–specific founder variants.

Due to the limited number of observed variants, HCM genes encoding components of the thin filament (*ACTC1*, *TNNC1*, *TNNI3*, *TNNT2*, and *TPM1*) and myosin light chain (MLC: *MYL2* and *MYL3*) were combined into groups based on their similar function. Nontruncating variants in MLC genes were significantly enriched in Egypt HCM cases vs. controls (*P* = 1.3 × 10^−7^) in contrast to the UK cohort (*P* = 0.35) with an Egypt case excess of 5.25% (*Table 2* and *Figure 1B*).

Six distinct variants were identified in each of *MYL2* (five missense and one splice site) and *MYL3* (six missense) (see Supplementary material online, *Table S2*). Of these, three *MYL2* variants were recurrently observed in Egyptian patients [c.173G > A (p.Arg58Gln) (*n* = 2, 0.39%); c.450G > T (p.Leu150Phe) (*n* = 3, 0.58%); and c.278C > T (p.Ala93Val) (*n* = 5, 0.97%)]. In *MYL3*, 3/6 missense variants observed in Egyptian patients were also recurrent [c.460C > T (p.Arg154Cys) (*n* = 2, 0.39%); c.518T > C (p.Met173Thr) (*n* = 4, 0.78%); and c.508G > C (p.Glu170Gln) (*n* = 6, 1.17%)]. With the exception of the known pathogenic *MYL2* c.173G > A (p.Arg58Gln) variant, these recurrent variants were absent from both the UK HCM and LMM/OMGL HCM cohorts. Also, the *MYL2* variant c.450G > T (p.Leu150Phe) has not been previously reported whereas the other 10 MLC variants are currently classified as VUS in ClinVar.

Regarding minor nonsarcomeric HCM genes, rare variants in both *CSRP3* (truncating and nontruncating) and *JPH2* (nontruncating) are more prevalent in the Egypt HCM cohort, as seen in both the case excess (*Figure 1B*) and proportion of genotype-positive case comparisons (*Figure 1C* and *Table 2*). However, as these variant classes are rarely detected in cases (1%–3%), the enrichment in Egypt cases over controls was not significant with Bonferroni correction (see Supplementary material online, *Table S6*).

To explore why variants in some minor HCM genes seem to be more prevalent in the Egypt HCM cohort compared with UK, we then examined the effect of consanguinity, and the frequency of homozygous variants, on this cohort.

### High prevalence of homozygous variants in the Egypt hypertrophic cardiomyopathy cohort

In the Egypt HCM cohort, 28% of patients with available data reported to have consanguineous parents. This prompted us to evaluate the frequency of homozygous variants, both for rare variants (FAF_popmax_ ≤ 4.0 × 10^−5^) and low-frequency variants sufficiently rare to cause biallelic disease (FAF_popmax_ ≤ 0.00126).^27^

As expected, homozygous variants were significantly more common in Egyptian patients compared with UK, for both the rare [4.1% (*n* = 21) vs. 0.1% (*n* = 1), *P* = 2 × 10^−7^] and low-frequency [2.1% (*n* = 11) vs. 0%, *P* = 8.5 × 10^−5^] variants. Homozygous variants in these genes were absent from Egypt and UK controls. Among Egypt HCM patients carrying homozygous variants, 31.3% reported not having consanguineous parents (i.e. first cousins), which may indicate an underreporting of consanguinity or more distantly related patients (*Table 3*). However, our analysis showed that patients with self-reported consanguinity were more likely to have homozygous variants compared with patients with reported nonconsanguinity [14% (14/99) vs. 4% (10/249), *P* = 0.0014]. The proportion of patients with missing family data carrying homozygous variants was 4.8% (8/166).

**Table 3:** Details of homozygous variants identified in the Egypt HCM cohort (*n* = 514) compared to Egypt controls (*n* = 400)

Gene	Variant type	cDNA variant	Protein variant	gnomAD FAF_popmax_	Control hom freq (count)	Control het freq (count)	HCM hom case freq (count)	HCM het case freq (count)
Sufficiently rare variants (gnomAD FAF_popmax_ ≤ 4 × 10^−5^) to cause monoallelic HCM								
*MYH7*	Missense	c.5122G > A	p.Glu1708Lys	0	0%	0%	0.19% (1)	0%
	Missense	c.4258C > T	p.Arg1420Trp	3.0 × 10^−6^	0%	0%	0.19% (1)	0.39% (2)
	Missense	c.3622G > C	p.Asp1208His	0	0%	0%	0.19% (1)	0.58% (3)
	Missense	c.1064C > T	p.Ala355Val	0	0%	0%	0.19% (1)	0%
	Missense	c.925G > A	p.Asp309Asn	1.1 × 10^−5^	0%	0%	0.19% (1)	0.78% (4)
	Frameshift^a^	c.5769delG	p.Ser1924AlafsTer9	0	0%	0%	0.19% (1)	3.11% (16)
*MYBPC3*	Missense	c.2908C > T	p.Arg970Trp	0	0%	0.25% (1)	0.19% (1)	0.39% (2)
	Missense	c.2458C > G	p.Arg820Gly	0	0%	0%	0.19% (1)	0%
	Inframe Deletion	c.2441_2443delAGA	p.Lys814del	0	0%	0%	0.19% (1)	0%
	Nonsense	c.1558G > T	p.Glu520Ter	0	0%	0%	0.19% (1)	0%
	Splice acceptor	c.1227-2A > G		0	0%	0%	0.19% (1)	0.19% (1)
*MYL2*	Missense	c.278C > T	p.Ala93Val	0	0%	0%	0.19% (1)	0.78% (4)
*MYL3*	Missense	c.508G > C	p.Glu170Gln	0	0%	0%	0.58% (3)	0.58% (3)
*TNNI3*	Missense	c.485G > T	p.Arg162Leu	0	0%	0%	0.19% (1)	0%
*CSRP3*	Splice acceptor	c.415-1G > C		0	0%	0%	0.19% (1)	0%
	Splice donor	c.414 + 1G > T		0	0%	0.25% (1)	0.58% (3)	0.19% (1)
	Missense	c.365G > A	p.Arg122Gln	2.9 × 10^−6^	0%	0%	0.19% (1)	0.58% (3)
Sufficiently rare variants (gnomAD FAF_popmax_ ≤ 0.00126) to cause biallelic HCM								
*MYBPC3*	Missense	c.3676C > T	p.Arg1226Cys	6.4 × 10^−5^	0%	0%	0.19% (1)	0%
	Missense	c.2618C > A	p.Pro873His	9.3 × 10^−5^	0%	0%	0.19% (1)	0%
	Missense	c.1321G > A	p.Glu441Lys	2.0 × 10^−4^	0%	2.0% (8)	0.39% (2)	4.3% (22)
*MYL3*	Missense	c.530A > G	p.Glu177Gly	4.4 × 10^−5^	0%	0.75% (3)	0.78% (4)	0.19% (1)
	Missense	c.170C > A	p.Ala57Asp	4.0 × 10^−4^	0%	0%	0.39% (2)	0.19% (1)
*JPH2*	Missense	c.572C > G	p.Pro191Arg^b^	2.0 × 10^−4^	0%	0.25% (1)	0.19% (1)	0.19% (1)

Summary of Egyptian variants that are sufficiently rare (gnomAD FAF_popmax_ ≤ 4 × 10^−5^) to cause monoallelic disease and additional variants that are sufficiently rare (gnomAD FAF_popmax_ ≤ 0.00126) to cause biallelic disease are also shown.

^a^The *MYH7* variant c.5769delG was caused by a nucleotide deletion that was predicted to ultimately give rise to a near full-length protein (i.e. only four AAs shorter than the wild type). The control frequency represents heterozygous counts (no homozygous variants were identified).

^b^This variant is unlikely to be pathogenic as it cooccurred with the pathogenic *MYH7* variant p.Arg870His in the HCM patient (also, its allele frequency in gnomAD-Ashkenazi Jewish population is 0.007215).

Two Egyptian patients presented with homozygous *MYBPC3* truncating variants [c.1558G > T (p.Glu520Ter) and c.1227-2A > G]. Truncating variants in *MYBPC3* are known to lead to severe, early-onset disease when expressed in homozygosity.^30^ The patient carrying the nonsense variant p.Glu520Ter was diagnosed with HCM at birth and died suddenly at the age of 11. In contrast, the patient carrying the c.1227-2A > G splice variant presented with HCM in adulthood. Accordingly, this splice acceptor variant is predicted (by SpliceAI) to result in an exon extension of 33 intronic bases into the *MYBPC3* transcript, which suggests that it maintains the open reading frame and may therefore function as an inframe insertion (see Supplementary material online, *Figure S3*).

Homozygous variants were particularly enriched in three minor HCM genes—*CSRP3* and the MLC genes *MYL2* and *MYL3*^18^ (*Table 3*). The proportion of rare homozygous variants was consistent across the major HCM genes, with 5%–7% of variants being homozygous in *MYH7*, *MYBPC3*, and thin filament genes (*Table 4* and *Figure 1D*). In contrast, MLC and *CSRP3* genes showed a substantially higher proportion of homozygous variants (25.6% and 33.3%, respectively). It is noteworthy that the increase in frequency of MLC homozygous variants from 14.3% (using the rare frequency threshold) to 25.6% (using the biallelic frequency threshold) was largely due to a recurrently observed low-frequency *MYL3* variant c.530A > G (p.Glu177Gly) in the Egyptian cohort (0.78%, *n* = 4).

**Table 4:** Relative contribution of homozygous variants for validated autosomal dominant HCM genes

	Variants with gnomAD FAF_popmax_ ≤ 4 × 10^−5^		Additional variants with gnomAD FAF_popmax_ ≤ 0.00126		Total hom freq (count/total)
Gene/gene groups	Total no. of patients	Hom case freq (count)	Total no. of patients	Hom case freq (count)	
*MYH7*	99	6.1% (6)	5	0%	5.8% (6/104)
*MYBPC3*	85	5.9% (5)	45	8.9% (4)	6.9% (9/130)
*CSRP3*	15	33.3% (5)	0	0%	33.3% (5/15)
*Myosin light chain*	28	14.3% (4)	11	54.5% (6)	25.6% (10/39)
*Thin filament*	18	5.6% (1)	8	0%	3.8% (1/26)
*ACTN2*	12	0%	8	0%	0% (0/20)
*JPH2*	14	0%	4	25% (1)	5.6% (1/18)
*PLN*	0	0%	0	0%	0%

For genes with an excess burden of rare variants in Egypt HCM patients vs. UK (*Table 2*). The relative contribution of rare (gnomAD FAF_popmax_ ≤ 4 × 10^−5^) homozygous variants under dominant HCM model (left) as well as of additional variants under recessive inheritance model (gnomAD FAF_popmax_ ≤ 0.00126) (right) are shown. MLC comprises *MYL2* and *MYL3*. Thin filament comprises *ACTC1*, *TNNC1*, *TNNI3*, *TNNT2*, and *TPM1*.

These findings explain the relatively higher proportion of rare variants in MLC and *CSRP3* genes observed in Egyptian patients compared with UK (*Figure 1*), with high consanguinity rates enabling variants to occur in a more penetrant homozygous state.

### A significantly higher proportion of variants of uncertain significance is observed in the Egypt hypertrophic cardiomyopathy cohort compared with UK hypertrophic cardiomyopathy

UK HCM patients are more likely to carry a disease-causing variant that has been previously well-characterized. Therefore, we hypothesize that in patients with rare variants in HCM genes, Egyptians have a higher proportion of VUSs compared with the UK HCM cohort.

**Figure 2:**
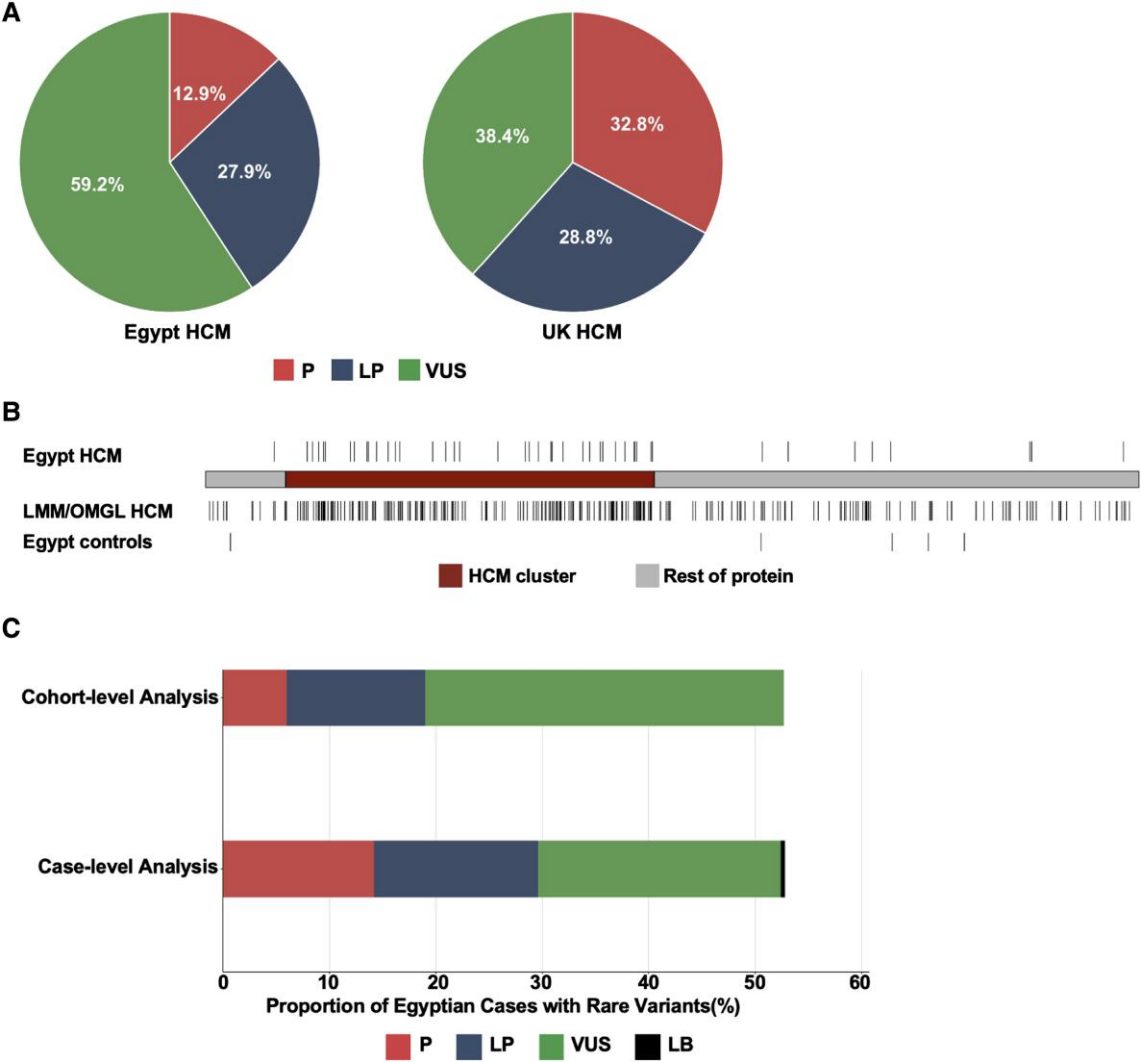
Incorporation of methods that compare frequencies between Egyptian patients and controls increases the yield of clinically actionable variants observed in the understudied population. (*A*) For patients with rare variants in validated hypertrophic cardiomyopathy genes, the distribution of rare variants by variant classification (pathogenic, likely pathogenic, and variant of uncertain significance) is shown. For patients with multiple hits (see Supplementary material online, *Tables S8* and *S9*), the variant with the highest pathogenicity was prioritized (i.e. pathogenic then likely pathogenic then variant of uncertain significance). The proportion of variants of uncertain significance was significantly higher in Egypt hypertrophic cardiomyopathy patients vs. UK (59.2% vs. 38.4%, *P* = 1.6 × 10^−5^). (*B*) Similar distribution of rare, missense *MYH7* variants between the Egypt- and large-scale hypertrophic cardiomyopathy cohort of majority European ancestry (Partners Laboratory of Molecular Medicine and Oxford Medical Genetics Laboratory, *n* = 6112) in the predefined hypertrophic cardiomyopathy cluster (red) (residues 167–931). Rare variants in the hypertrophic cardiomyopathy cluster were enriched in the Egypt hypertrophic cardiomyopathy cohort over Egypt controls with an etiological fraction of 0.99. (*C*) Application of modified variant interpretation guidelines increases the proportion of Egypt hypertrophic cardiomyopathy patients with clinically actionable variants from 19% to 29.6%. Rare variants were initially classified based on the standard American College of Medical Genetics and Genomics/Association for Molecular Pathology guidelines (initial analysis) and then reclassified after integrating ancestry-matched controls into the analysis (cohort-informed analysis). HCM, hypertrophic cardiomyopathy; P, pathogenic; LP, likely pathogenic; VUS, variant of uncertain significance; LMM/OMGL, Partners Laboratory of Molecular Medicine and Oxford Medical Genetics Laboratory; LB, likely benign.

The proportion of patients with rare variants in HCM genes was 46.7% (*n* = 240) and 28.9% (*n* = 198) in the Egypt and UK HCM cohorts, respectively. The proportion of VUSs was significantly higher in the Egypt HCM cohort compared with UK (59.2% vs. 38.4%, *P* = 1.6 × 10^−5^) (*Figure 2A*).

### Analysis of hypertrophic cardiomyopathy rare variation in the ancestry-matched Egyptian case–control cohort increases the proportion of clinically actionable variants

The availability of genetic data from ancestry-matched patients and controls analysed here enabled us to reevaluate the classification of identified VUSs in patients. Based on its presence in two Egyptian controls, we were able to reclassify 1/104 VUSs [*MYBPC3* c.305C > T (p.Pro102Leu)] to likely benign. Also, the detection of some variants in multiple Egyptian cases, along with absence in controls, provides additional evidence for pathogenicity and allowed to reclassify 1 VUS to LP (0.96%) and 10/30 LP variants to P (33.3%) (see Supplementary material online, *Methods* and *Table S2* for details).

In addition to the enrichment of specific variants in HCM cohorts, variants within highly pathogenic regions of disease genes can also have a high likelihood of pathogenicity. We previously identified specific regions in sarcomeric genes in which rare nontruncating variants were significantly enriched in HCM patients of majority European ancestry (LMM/OMGL) compared with controls.^13^ For variants residing in these case-enriched clusters, the probability that a rare nontruncating variant identified in an individual with HCM is disease-causing can be quantified through case–control analysis (defined by the EF). We confirmed that rare missense variants in the previously identified HCM cluster of *MYH7* (residues 167–931) were similarly enriched in the Egyptian HCM cases as in the majority European-ancestry cases (Egypt HCM: 12.45% vs. Egypt control: 0%, *P* = 2.2 × 10^−16^) (*Figure 2B*). This allowed us to upgrade the classification of additional 23 *MYH7* VUSs to LP and three *MYH7* LP variants to P, which increased the overall proportion of clinically actionable distinct *MYH7* missense variants from 36% to 84% (see Supplementary material online, *Methods* for details).

Taken together, applying the above-mentioned approaches to variant interpretation increased the overall proportion of Egypt HCM patients with clinically actionable variants from 19% to 29.6% (relative increase from 40.8% to 60.4% in the patient subgroup with rare variants in validated HCM genes) (*Figure 2C*).

### Enrichment of nonrare variants in Egypt hypertrophic cardiomyopathy patients

A growing body of evidence suggests that low-frequency variants could contribute to disease risk with intermediate effects.^31^ Therefore, we sought to assess if low-frequency variants (i.e. 4 × 10^−5^ < gnomAD FAF_popmax_ < 0.01) were enriched in the Egypt HCM cohort compared with controls. Twenty nonrare protein-altering variants in validated HCM genes were observed in more than one patient in the Egypt HCM cohort (see Supplementary material online, *Table S11*). Of these, *MYBPC3* c.1321G > A (p.Glu441Lys) and *TNNT2* c.832C > T (p.Arg278Cys) were nominally enriched in Egyptian patients over controls [4.67% vs. 2%, *P* = 0.03, OR 2.4 (95% CI: 1.0–6.2) and 1.17% vs. 0%, *P* = 0.04, OR 10.2 (95% CI: 0.6–182.3), respectively] (although not statistically significant after multiple testing correction). While p.Arg278Cys is enriched in HCM in both Egyptian and European-ancestry cohorts, including the analysed UK cohort, p.Glu441Lys is infrequently observed in most European HCM cohorts (see Supplementary material online, *Table S12*).^32–35^ Several publications reported the frequent cooccurrence of these variants with other sarcomeric variants suggesting that they may act as disease modifiers.^31,36–39^ Notably, 37.5% (*n* = 9/24) and 33.3% (*n* = 2) of Egyptian patients with p.Glu441Lys and p.Arg278Cys, respectively, carried another rare sarcomeric variant, including two patients who carried p.Glu441Lys in homozygosity (see Supplementary material online, *Table S12*). In addition, we investigated the prevalence of the common *BAG3* missense variant c.451T > C (p.Cys151Arg) in the Egypt HCM cohort, which was recently reported as one of the two most strongly associated loci for HCM in a European-ancestry genome-wide association study (GWAS) data set.^40^ The OR of p.Cys151Arg was highly similar between the Egyptian cohorts and the published European-ancestry data sets, 1.47 (95% CI: 1.12–1.95) and 1.42 (95% CI: 1.29–1.55), respectively,^40^ although the population frequency for the minor risk allele is lower in Egypt (control MAF = 0.12) than Europeans (gnomAD-non-Finnish European MAF = 0.22).

### Higher prevalence of *TRIM63* biallelic variants in the Egypt hypertrophic cardiomyopathy cohort compared with Europeans

We identified seven homozygous and one compound heterozygous *TRIM63* variants in the Egypt HCM cohort (total = 8/374, 2.14%) (see Supplementary material online, *Table S13*). No biallelic *TRIM63* variants were identified in the Egypt control cohort. Similar to the recent study, c.224G > A (p.Cys75Tyr) (1.34%) and c.739C > T (p.Gln247Ter) (0.53%) (both homozygous) were the most frequently identified *TRIM63* biallelic variants in the Egypt HCM cohort.^41^ The missense variant Cys75Tyr was observed in heterozygosity in 0.5% (2/400) of Egyptian controls and in two individuals from the North African subpopulation of the Great Middle Eastern variome study (in hetero- and homozygosity).^42^ A markedly higher frequency of biallelic *TRIM63* variants was observed in the Egypt HCM cohort compared with both the analysed UK HCM cohort and the recently published European-ancestry enriched cohort (2.14% vs. 0.16%, *P* = 0.0019, and vs. 0.4%, *P* = 5.8 × 10^−5^, respectively).^41^

## Discussion

This study represents the largest and most comprehensive analysis of HCM in the MENA region to date. We show that cohort-level analysis of rare variation in the hitherto understudied Egyptian population provides accurate interpretation of variants rarely observed in published HCM patient groups. In addition, we demonstrate that studying consanguineous populations allows for distinguishing between the relative pathogenicity and penetrance of disease-causing genes (*Structured Graphical Abstract*).

First, we show that the proportion of patients with rare variants in validated HCM genes was higher in the Egypt HCM cohort compared with UK HCM (52.7% vs. 32.0%). The relatively higher burden of rare variants identified in Egyptian patients was still observed after excluding Egyptian patients with consanguineous parents (Egypt HCM 48.5% vs. UK HCM 32.0%), which suggests that the difference in the genetic profiles between both ancestral cohorts is not primarily driven by consanguinity. Recent data from SHaRe registry demonstrated that patients carrying sarcomere variants presented with HCM at an earlier age and had a worse prognosis.^10^ Indeed, Egyptian patients analysed in this study presented with a markedly younger age of onset (by ∼20 years) and more severe LVH compared with UK HCM, which is reflected in the higher genetic yield. It is noteworthy that Egyptian patients referred to AHC are typically severely affected with HCM prior to admission compared with other equivalent cohorts. To account for a possible referral bias, we also compared the proportion of genotype-positive patients attributable to each gene between both ancestral cohorts.

As expected, *MYBPC3* and *MYH7* accounted for the majority of rare variation. However, some key differences are observed between the Egypt and UK HCM cohorts. For example, we have recently reported the identification of a new class of pathogenic variants in HCM: a nonsense-mediated decay-incompetent frameshift variant (c.5769delG, p.Ser1924AlafsTer9) in *MYH7*, which was recurrently observed in Egyptian patients (3.3%) and absent from large-scale HCM patients of majority European ancestry.^16,18^

The main unique finding, however, relates to the clear prevalence of HCM minor genes, particularly *MYL2*, *MYL3* (MLC), and *CSRP3*, in the Egyptian population and the enrichment of homozygous variants in these genes. A noticeably higher proportion of rare variants in MLC genes was observed in the genotype-positive Egypt HCM cohort compared with UK HCM (10.33% vs. 2.28%), including five recurrent variants that were absent from the latter cohort. Interestingly, a substantial proportion of MLC variants (25.6%) were observed in homozygosity, suggesting such variants are less penetrant in heterozygosity. In agreement with this notion, Claes *et al.*^43^ demonstrated that the expression of the Dutch founder *MYL2* missense variant c.64G > A (p.Glu22Lys) in isolation was associated with low HCM penetrance and a benign disease manifestation and that the presence of an additional risk factor, including the cooccurrence of another sarcomeric variant, increased the disease penetrance from 36% to 89% in *MYL2*-variant carriers. In addition, the authors reported that patients carrying p.Glu22Lys in homozygosity were diagnosed with end-stage heart failure due to HCM at the ages of 30 and 31, respectively.^43^ It is therefore plausible to conclude that the high consanguinity rates observed in the Egypt HCM cohort enabled MLC variants to occur in a more penetrant homozygous state.

This may explain, at least in part, the relatively higher proportion of rare MLC variants observed in the analysed consanguineous Egypt HCM cohort compared with UK. While biallelic truncating variants in *MYL2/MYL3* are associated with severe early-onset cardioskeletal myopathies, only two homozygous missense variants in *MYL3* have previously been reported—c.427G > A (p.Glu143Lys)^44,45^ and c.170C > A (p.Ala57Asp).^46,47^ It is noteworthy that homozygous expression of the nonrare variant c.170C > A (p.Ala57Asp), which was observed in HCM patients of Iranian and Tunisian origins born to consanguineous parents,^46,47^ was also observed in the Egypt HCM cohort (in two homozygous and one heterozygous carrier). Homozygous patients presented with HCM at a relatively younger age (21.5 + 14.8 vs. 34 years) and had a greater left ventricular maximal wall thickness (LVMWT) (27 + 2.8 mm vs. 16 mm) compared with the heterozygous carrier. Although a previous functional study with induced pluripotent stem cell-derived cardiomyocytes (iPSC-CMs) did not demonstrate a phenotype for this variant in either the heterozygous or homozygous state,^48^ the fact that is has now been detected as a homozygous variant in four HCM cases (and absent from controls) suggests it is contributing to HCM.

Similar to MLC-encoding genes, variants in *CSRP3* were more likely to present in homozygosity (33.3%) than the major sarcomere-encoding HCM genes (*MYH7*, *MYBPC3*, and thin filament genes, 5%–7%). Two of the three homozygous *CSRP3* variants were truncating (c.415-1G > C and c.414 + 1G > T). These variants were observed in one and three Egyptian patients, respectively. Patients carrying these variants presented with HCM at an average age of 45.5 ± 8.7 and a LVMWT of 16.5 ± 2.4 mm. The variant c.414 + 1G > T was also observed in heterozygosity in one Egyptian control and HCM patient, respectively. The control did not carry any other rare HCM-associated variants whereas the HCM patient carried the pathogenic *MYH7* c.5769delG (p.Ser1924AlafsTer9) variant and the *JPH2* VUS c.296G > A (p.Ser99Asn), suggesting that in the heterozygous state, this variant requires an additional mutational burden to express the HCM phenotype. To date, only three HCM patients carrying biallelic truncating *CSRP3* variants have been reported in Poland and France, two of whom were confirmed to be born to consanguineous parents.^49,50^ These patients were diagnosed with HCM at the age of 18, 24, and 54 years, respectively. Together, these studies suggest that expression of biallelic *CSRP3* truncating variants, in contrast to the severe early-onset phenotypes observed with biallelic truncating variants in genes like *MYBPC3* and *ALPK3*, is associated with more typical adult-onset HCM, despite the fact that most reports for *CSRP3*-associated HCM involve heterozygous variants and dominant inheritance. These reports demonstrated that *CSRP3* belongs to a limited number of nonsarcomeric genes with a primary pathogenic role in HCM, which collectively account for <2% of cases.^21^

Taken together, these findings demonstrate that this study is uniquely able to examine the frequency of homozygous variants in dominant HCM genes using a large consanguineous cohort to inform the relative penetrance of variants in these genes. The recurrence of these variants in the Egyptian cohort, which may indeed have generally higher prevalence in other consanguineous populations of the MENA region, exemplifies the need to integrate new guidelines tailored to the classification of homozygous variants into the current ACMG framework in order to enhance the utility of clinical genetic testing.

The observed high prevalence of consanguinity in the Egyptian cohort analysed in this study has also enabled us to validate recent findings reporting the association of the recessive gene *TRIM63* to HCM.^41^ Indeed, the prevalence of biallelic *TRIM63* variants was markedly higher in Egyptian patients compared with HCM patients of majority European ancestry suggesting that recessive inheritance patterns of HCM are more likely to occur in consanguineous populations.

Our data highlight the value of performing ancestry-matched case–control analysis coupled with standardized guidelines and quantitative approaches to variant interpretation to accurately classify variants observed in underrepresented populations. We demonstrate that these approaches enabled 23.1% of distinct VUSs identified in the Egypt HCM cohort to progress to LP, enabling a more equitable application of clinical genetic testing in the region. Expanding the MENA data set in resources like gnomAD will further improve the interpretation of VUSs by providing more accurate frequencies of these variants in the population. In addition, long-term monitoring of family members carrying the same VUS for the investigation of cosegregation provides another opportunity for accurate variant interpretation.^51^

It is noteworthy that despite the relatively severe phenotypes observed in our cohort, almost 50% of HCM patients in this study presented with an unexplained genetic aetiology. This finding is not unexpected as recent evidence suggests that the genetic aetiology of HCM is more complex and that common variants with individually small (but collectively large) effect sizes could explain some of the missing heritability of HCM.^40,52^

We also analysed the prevalence of ‘non-Mendelian’ variants, mainly low-frequency variants in the genes associated with monogenic HCM, which revealed that *MYBPC3* c.1321G > A (p.Glu441Lys) and *TNNT2* c.832C > T (p.Arg278Cys) were nominally enriched in Egyptian patients. Intriguingly, p.Glu441Lys is clearly more prevalent in the Egypt HCM cohort, suggesting that it could be more relevant to Egyptian patients.^32–35^ More comprehensive genetic and functional studies focusing on these and other low-frequency variants are required to elucidate their effect on the HCM phenotype. In addition, we assessed the association of the recently reported common HCM *BAG3* missense variant c.451T > C (p.Cys151Arg) with disease in the Egyptian cohort and showed that the OR of p.Cys151Arg was highly similar compared with published European data sets.^40^ This suggests that there will be likely many shared loci between both populations, although well-powered GWAS in MENA populations are warranted to accurately quantify HCM PRS for future clinical use.

A limitation of the study is that the targeted gene panel used does not include genes that have been recently shown to be associated with HCM (*ALPK3*, *FHOD3*, and *FLNC*).^53–57^ Also, the study design did not account for common and copy number variations, noncoding variants, or epigenetic factors, which may contribute to HCM pathogenesis. Finally, the lack of large-scale population reference data sets from the MENA region poses challenges for the practice of clinical genetics in these populations, including the Egyptian HCM cohort analysed in this study. Until these data sets become publicly available, it is crucial to address the major issue of detecting substantial proportions of VUS in patients by utilising data from moderately sized, ancestry-matched controls as shown in this study. For variant classes highly enriched in case cohorts, there is a low likelihood that variants identified in patients and absent in existing control data sets will be shown to be actually nonrare in the population. However, the risk of false positive results due to the absence of large population data sets means that novel variants identified in patients should be cautiously interpreted.

In conclusion, we have demonstrated that detailed analysis of rare variation in validated HCM genes in underrepresented populations reveals novel findings with relevance to both clinical genetic testing and our understanding of the genetic aetiology of HCM. We report the contribution of minor HCM genes, rarely observed in outbred populations, to disease pathogenicity. We also show a higher prevalence of biallelic *TRIM63* variants in Egyptian patients suggesting that recessive inheritance of HCM is more likely to occur in consanguineous populations. These unique genetic features exemplify the value of studying diverse ancestral populations. The highly consanguineous Egypt HCM cohort opens new research avenues for the discovery of novel recessive genes in future exome or genome sequencing studies.

## Supplementary Material

ehad372_Supplementary_Data

## Data Availability

The data underlying this article are available in the article and in its online supplementary material.

## Pre-registered Clinical Trial Number

None supplied.
