# Supplementary material for: Ethnicity, consanguinity, and genetic architecture of hypertrophic cardiomyopathy
Source: Eur Heart J. 2023 Jul 11;44(48):5146–58. doi: 10.1093/eurheartj/ehad372 (PMC10733735; doi:10.1093/eurheartj/ehad372)
Supplement: ehad372_Supplementary_Data [file ehad372_supplementary_data.zip › Supplementary Methods_revised version_Feb2023.docx]

**Supplementary Methods**

### Rare variant filtering and classification

Rare variants in validated HCM genes were defined as having a filtering allele frequency (FAF) of ≤ 4x10^-5^ in gnomAD based on the statistical framework proposed by Whiffin *et al.,* 2017^1^. In this study, WES data was downloaded from the gnomAD database (https://gnomad.broadinstitute.org; version v2.1.1) and only variants with a PASS filter were included in the analysis. Variants in the sarcomere genes (*ACTC1*, *MYBPC3*, *MYH7*, *MYL2*, *MYL3*, *TNNC1*, *TNNI3*, *TNNT2* and *TPM1*) and the minor HCM genes (*PLN* and *CSRP3*) were classified into Pathogenic (P), Likely Pathogenic (LP) and Uncertain Significance (VUS) using CardioClassifier, which integrates the ACMG/AMP guidelines to aid in the interpretation of variants identified in a gene associated with an inherited cardiac condition ^2,3^. The following rules are automatically activated by CardioClassifier:

- **PM1:** Mutational hotspot or well-studied functional domain without benign variation. This rule was applied at moderate level to *MYH7* missense variants located in the mutational hotspot (residues 181-937).
- **PM2:** Low frequency in population databases (i.e., FAF in ExAC < 4×10^−5^). In this analysis, gnomAD was used as the reference population, so gnomAD data (https://gnomad.broadinstitute.org; version v2.1.1) was downloaded for the validated HCM genes. Then, the gnomAD FAF_popmax_ was then calculated using the R script “frequencyFilter” available in github (<https://github.com/ImperialCardioGenetics/frequencyFilter/blob/master/src/precompute_exac_af_filter.R>).
- **PVS1:** Truncating variants in genes *CSRP3*, *FHL1*, *MYBPC3*, *TNNI3*, *TNNT2* and *PLN*, which are statistically enriched in LMM/OMGL patients over controls.
- **PS4:** Variant is statistically enriched in LMM/OMGL patients over controls with the rule activated if the patient count was > 2 and the Fisher’s exact test p-value was < 1.79 × 10^−6^ (Bonferroni correction).
- **PM4:** Protein length changing variants as a result of inframe insertion/deletions or stop lost.
- **PP3:** Missense variant with multiple lines of computational evidence supporting a deleterious effect. The computational prediction algorithms include: SIFT, PolyPhen2 var., LRT, Mutation Taster, Mutation Assessor, FATHMM, CADD and Grantham scores. This rule is activated if at least 5/8 tools predict a deleterious effect, with only 1 tool predicting benign and <3 with “unknown” classifications or if >3 tools have unknown variant classifications while all other tools predict a deleterious effect.
- **PM5/PS1:** Novel missense variant where a different missense variant at the same amino acid residue is classified as pathogenic or novel missense variants with the same amino acid change as an established pathogenic variant (PS1). Novel variants are defined as “pathogenic” if multiple submitters in ClinVar confer pathogenicity with no conflicting evidence.

*ACTN2* and *JPH2* are not included in CardioClassifier and thus were manually classified according to the ACMG/AMP guidelines.

Then, rare variants, which activate the PM2 rule, were manually curated using ClinVar and PubMed resources for evidence regarding the following ACMG/AMP rules:

- **PP1:** Co-segregation with disease (supporting ≥ 3 meiosis, moderate ≥ 5, strong ≥ 7)
- **PM2/PM6:** *De novo* inheritance (with/without confirmed maternity and paternity)
- **PS3:** rule relating to evidence of deleterious effects of variants was not applied as functional approaches are not well-validated for HCM variants^4^

### Principal Component Analysis (PCA) of the analysed Egyptian case and control cohorts

To confirm that Egyptian case and control cohorts were ancestry-matched, Egyptian data was projected on to the two major principal components of the HapMap populations (Supplementary Figure 2). The Egyptian data integrated in the principal component analysis (PCA) comprised common SNPs (mostly exonic SNPs in Inherited Cardiac Conditions’ (ICC) genes included in the TruSight Cardio Sequencing Panel). PCA was performed using PLINK software (version 1.9) and the data was visualised using R.


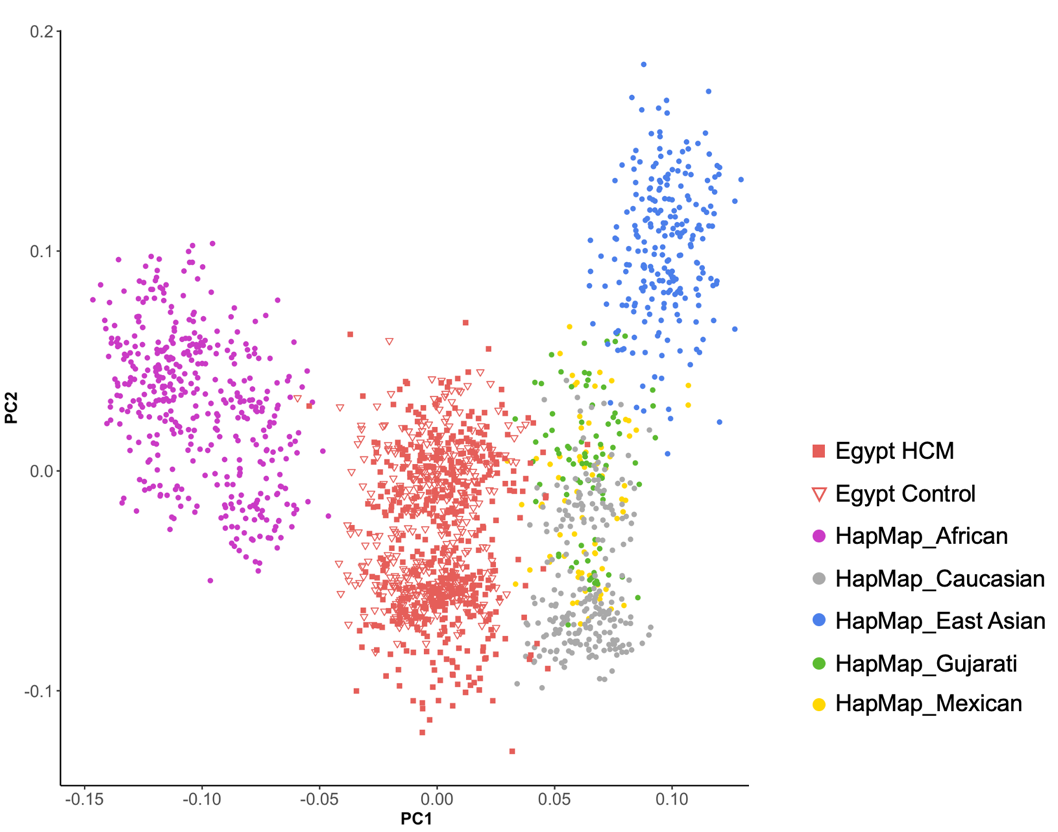


**Supplementary Figure 1: Principal Component Analysis (PCA) plot for common SNPs between the Egyptian (HCM and Control) and HapMap populations.** x-axis denotes the value PC1, while y-axis denotes the value of PC2, with each dot in the figure representing one individual. Egyptian individuals (red) include both Egypt HCM patients (squares) and Egypt controls (triangles). HapMap populations are colour-coded as shown in the legend. Both Egypt HCM patients and controls clusters overlap in one distinct cluster that is distinguishable from the HapMap populations which confirms that they are well matched to each other. A caveat of this PCA analysis is that it was performed using targeted panel data. Genome-wide data could reveal more accurate and distinct clustering. PC: principal component.

### “Rare” variants defined using gnomAD introduced bias in comparing HCM genetic architecture between Egypt and UK HCM cases

The definition of variant rarity in the Egyptian HCM cohort was based on five gnomAD populations: African/African American, Admixed American, East Asian, Non-Finnish European and South Asian while data from the MENA region was lacking in the dataset. Thus, variants considered “rare” in Egyptian cases may actually be common/non-rare in the general Egyptian population. To evaluate the bias in defining variant rarity based on gnomAD, the burden of rare synonymous variants in all ICC genes was compared between the Egyptian (i.e. Egyptian HCM cases + Egyptian controls) and the HCM cohort of majority European-ancestry (UK cases + UK controls) cohorts. Only synonymous variants were selected for this analysis as they are not expected to be disease-causing and thus provide an unbiased approach to analyse inflation. Burden testing data was evaluated for inflation with a quantile-quantile (Q-Q) plot and then the genomic inflation factor (GIF) was estimated as per the formula proposed by Guo *et al.* 2018^5^.

As depicted in Supplementary Figure 2, the resulting distribution of the ICC single-gene p-values was characterised by a GIF of 2.770. This finding reflects an inflation of the expected- (under uniform distribution) and observed p-values (from burden testing data) above the expected GIF = 1, suggesting the presence of bias.


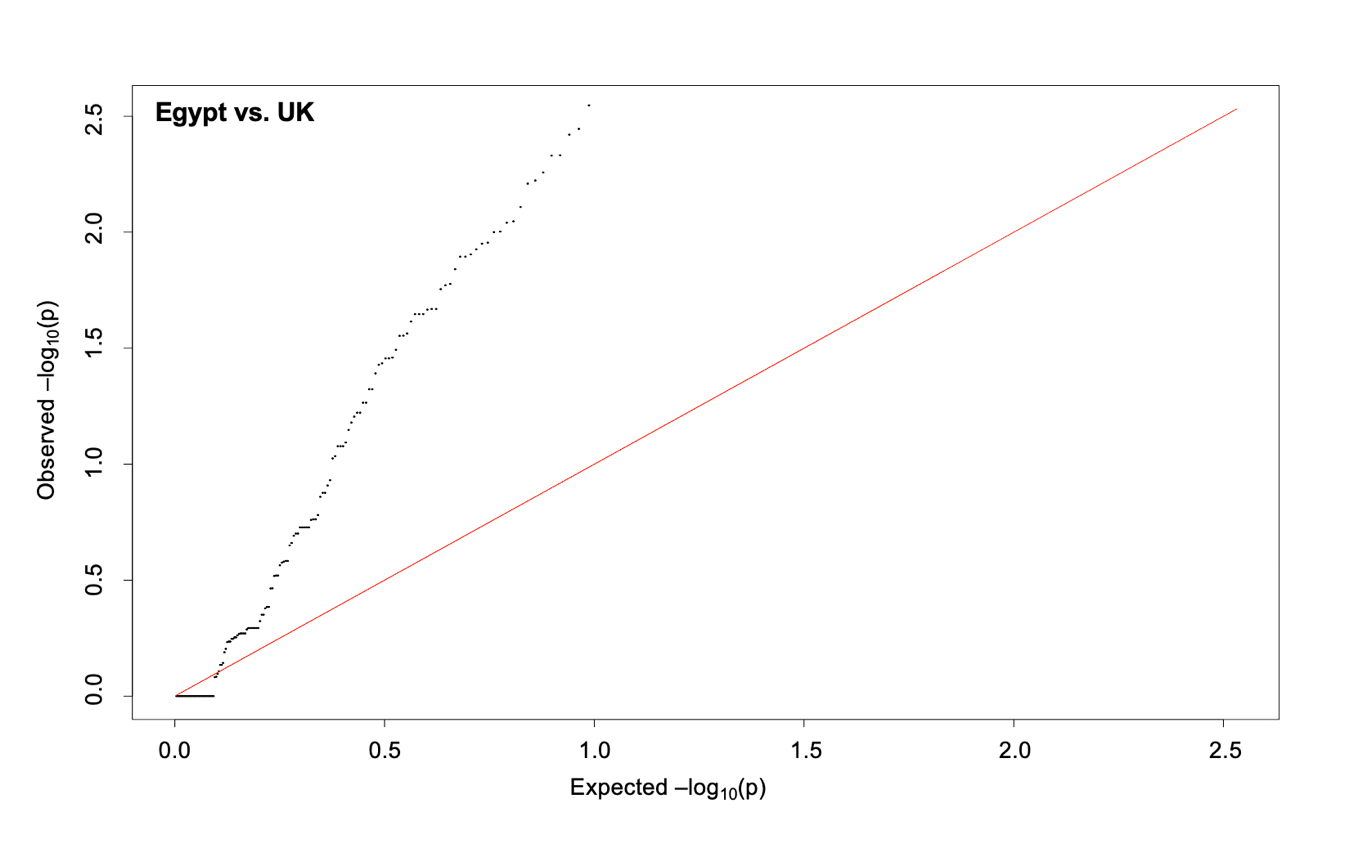
**Supplementary Figure 2:** **Quantile-quantile (Q-Q) plot of Egyptian vs. UK burden testing data based on synonymous variants.** Expected -log_10_ p-values for rare (gnomAD FAF_popmax_ ≤ 4x10^-5^) synonymous variants, under the uniform distribution (red line), were plotted against the observed -log_10_ p-values from the burden testing data for each ICC gene. The plot shows a discordance between expected and observed values with a genomic inflation factor (GIF) of 2.770.

The observed high GIF confirmed that the biased definition of variant rarity based on gnomAD confounded the comparison of rare variation between Egyptian and UK HCM cohorts. This bias might explain the observed higher frequency of rare variants in HCM genes in Egyptian HCM cases compared to UK (refer to Figure 1A in Manuscript).


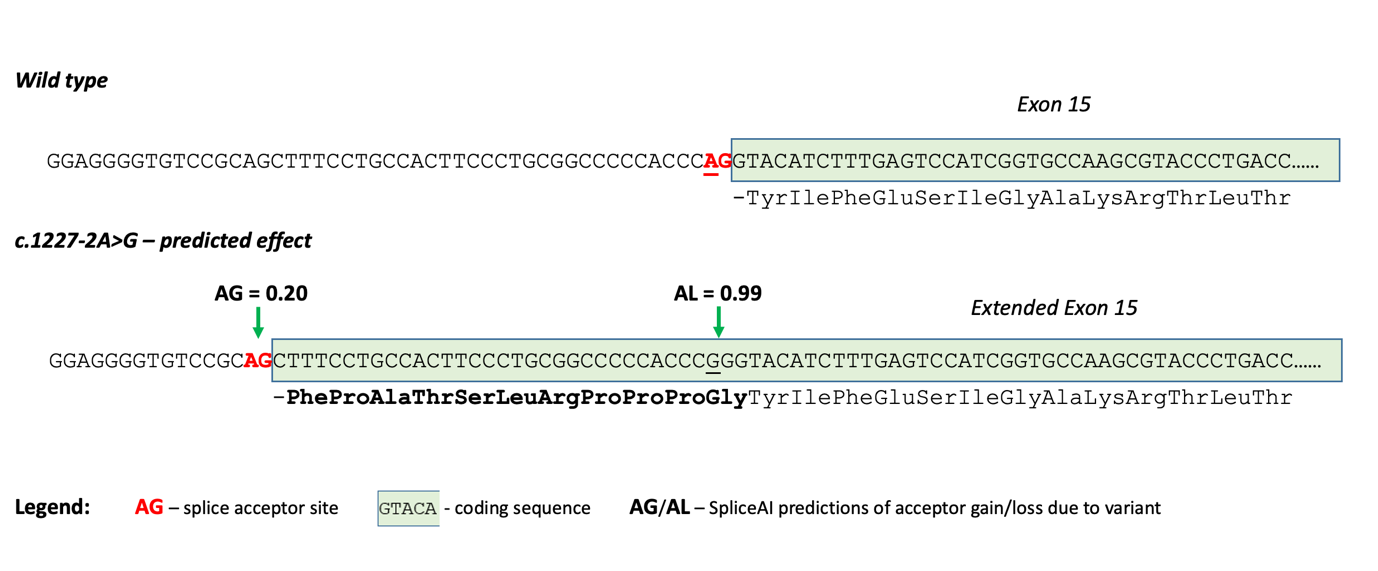


**Supplementary Figure 3: Schematic presentation of the predicted effect (by SpliceAI) of the splice acceptor variant c.1227-2A>G on the exonic sequence of M*YBPC3*.** The variant is predicted to result in an exon extension of 33 intronic bases into the *MYBPC3* transcript suggesting that it maintains the open reading frame and thus may function as an inframe insertion.

### Variant reclassification approaches applied to Egypt HCM cohort data

The classification of VUSs identified in the Egypt HCM cohort was re-evaluated by adopting three approaches. First, VUSs that were commonly observed in the ancestry-matched control cohort (i.e. n≥2; MAF≥0.0025) were reclassified to likely benign as per the ACMG/AMP rules BS1(*allele frequency is greater than expected for disorder)* and BP4 (*multiple lines of computational evidence suggest no impact on gene or gene product (conservation, evolutionary, splicing impact, etc.)*)^2^. Second, the modified ACMG/AMP PS4 rule (*prevalence of the variant in affected individuals is significantly increased compared with the prevalence in controls*) proposed by ClinGen was adopted, whereby variants prevalent among at least 2, 6 or 15 patients could activate the PS4- supporting, moderate and strong rules, respectively (Supplementary Table 2)^6^. The PS4 evidence strength levels associated with these specific patient counts were based on likelihood ratios with ideal target thresholds set by ClinGen of 10 (PS4-supporting), 30 (moderate) and 100 (strong), respectively^6^. Finally, the localisation of rare non-truncating sarcomeric variants to previously defined case-enriched HCM clusters was assessed^4^ . We previously identified specific regions in sarcomeric genes in which rare non-truncating variants were significantly enriched in >6,000 HCM cases of majority European-ancestry (denoted as LMM/OMGL) compared to controls^4,7^. For variants residing in these case-enriched clusters, the etiological fraction (EF) (defined as EF = (OR-1)/OR) was calculated as it provides a quantitative estimate of the probability that a rare non-truncating variant identified in an individual with HCM is disease-causing. VUS and LP variants located in an HCM cluster with an EF>0.95 were then reclassified to LP and P, respectively, as per the modified EF-based PM1 ACMG/AMP rule (*non-truncating variant in gene/protein region with EF ≥ 0.95*)^4^.

As the relative case-control frequencies can differ across population groups, similar enrichment needs to be demonstrated in the population of interest before adopting these modified guidelines. Given the abundance of rare missense variants observed in *MYH7* in the Egypt HCM cohort, the EF-based PM1 ACMG/AMP rule was validated for *MYH7* variants (there were insufficient variants to accurately assess clustering in the troponin genes). Also, a similar distribution of rare missense *MYH7* variants was observed between Egyptian and LMM/OMGL patients (Figure 2B). The distribution of missense *MYH7* variants was visualised in a lolliplot using the TrackViewer package (v 1.22.1) in R statistical environment (v 3.6.3). The EF for the pre-defined HCM cluster for *MYH7* missense variants (residues 167-931) was calculated using the Egypt control cohort as the reference population. 95% confidence intervals (CI) for EF values were calculated in R as described by Hildebrandt *et al.,* 2006^8^. The EF for Egyptian variants within the pre-defined HCM cluster was 0.99(95%CI:0.86-1.0), thus, the EF-based PM1_strong ACMG/AMP was applied on rare Egyptian *MYH7* missense variants, irrespective of prior variant classification (Supplementary Table 2). ­­

**References**

1. Whiffin N, Minikel E, Walsh R, O’Donnell-Luria AH, Karczewski K, Ing AY, et al. Using high-resolution variant frequencies to empower clinical genome interpretation. *Genetics in Medicine*. 2017;19:1151–8.
2. Richards S, Aziz N, Bale S, Bick D, Das S, Gastier-Foster J, et al. Standards and guidelines for the interpretation of sequence variants: a joint consensus recommendation of the American College of Medical Genetics and Genomics and the Association for Molecular Pathology. *Genetics in Medicine*. 2015;1:405–23.
3. Whiffin N, Walsh R, Govind R, Edwards M, Ahmad M, Zhang X, et al. CardioClassifier: disease- and gene-specific computational decision support for clinical genome interpretation. *Genetics in Medicine*. 2018;20:1246–54.
4. Walsh R, Mazzarotto F, Whiffin N, Buchan R, Midwinter W, Wilk A, et al. Quantitative approaches to variant classification increase the yield and precision of genetic testing in Mendelian diseases : the case of hypertrophic cardiomyopathy. *Genome Med.* 2019;11:1–18.
5. Guo M.H, Plummer L, Chan Y.M, Hirschhorn J.N. & Lippincott M.F. Burden Testing of Rare Variants Identified through Exome Sequencing via Publicly Available Control Data. *Am J Hum Genet*. 2018;103:522–534.
6. Kelly MA, Caleshu C, Morales A, Buchan J, Wolf Z, Harrison SM, et al. Adaptation and validation of the ACMG / AMP variant classification framework for MYH7 -associated inherited cardiomyopathies : recommendations by ClinGen’s Inherited Cardiomyopathy Expert Panel. *Genet Med*. 2018;20:351–359.
7. Walsh R, Thomson KL, Ware JS, Funke BH, Woodley J, McGuire KJ, et al. Reassessment of Mendelian gene pathogenicity using 7,855 cardiomyopathy cases and 60,706 reference samples. *Genetics in Medicine*. 2017;19:192–203.
8. Hildebrandt M, Bender R, Gehrmann U & Blettner M. Calculating confidence intervals for impact numbers. *BMC Med Res Methodol.* 2016;32:6.
